# Non-Markovianity of qubit evolution under the action of spin environment

**DOI:** 10.1038/s41598-019-39140-2

**Published:** 2019-02-27

**Authors:** Sagnik Chakraborty, Arindam Mallick, Dipanjan Mandal, Sandeep K. Goyal, Sibasish Ghosh

**Affiliations:** 1 Optics and Quantum Information Group, The Institute of Mathematical Sciences, C. I. T. Campus, Taramani, Chennai, 600113 India; 2 Homi Bhabha National Institute, Training School Complex, Anushakti Nagar, Mumbai, 400094 India; 3 The Institute of Mathematical Sciences, C. I. T. Campus, Taramani, Chennai, 600113 India; 4 Department of Physics, Indian Institute of Science Education and Research, Mohali, Punjab 140306 India

**Keywords:** Quantum information, Qubits

## Abstract

The question, whether an open system dynamics is Markovian or non-Markovian can be answered by studying the direction of the information flow in the dynamics. In Markovian dynamics, information must always flow from the system to the environment. If the environment is interacting with only one of the subsystems of a bipartite system, the dynamics of the entanglement in the bipartite system can be used to identify the direction of information flow. Here we study the dynamics of a two-level system interacting with an environment, which is also a heat bath, and consists of a large number of two-level quantum systems. Our model can be seen as a close approximation to the ‘spin bath’ model at low temperatures. We analyze the Markovian nature of the dynamics, as we change the coupling between the system and the environment. We find the Kraus operators of the dynamics for certain classes of couplings. We show that any form of time-independent or time-polynomial coupling gives rise to non-Markovianity. Also, we witness non-Markovianity for certain parameter values of time-exponential coupling. Moreover, we study the transition from non-Markovian to Markovian dynamics as we change the value of coupling strength.

## Introduction

We rarely come across systems that are completely isolated from the surrounding world. Had it been the case, dealing with quantum mechanical systems would have been lot more easier. So, although arduous to deal with, real quantum systems are mostly open quantum systems – a system interacting with an environment. In these situations, information exchange between system and environment becomes an essential feature. Information that has been previously transferred to the environment may come back and affect the system, and this may appear as a memory-effect on the system. When this information backflow from the environment is negligible we have a situation analogous to the discrete Markov process, where the instantaneous state of the system depends solely on the immediately previous step, the system dynamics is called *memory-less* or *Markovian*^1,2^. On the other hand, when this information backflow affects the system significantly i.e. when some long past history of the system influences its present state, the system dynamics becomes retentive, and is called *non-Markovian*.

In recent years, non-Markovianity has been used as a resource in a number of information theoretic protocols, namely, channel discrimination^3^, preserving coherence and correlation^4–19^ and retrieving quantum correlations in both quantum and classical environments^16,20–23^. Non-Markovian effects also play important roles in areas ranging from fundamental physics of strong fields^24,25^ to energy transfer process of photosynthetic complexes^26^.

Owing to its diverse applications, various aspects of non-Markovianity are now being studied. Lately, researchers have been focusing on transition from non-Markovian to Markovian dynamics^27–32^. Some of them have dealt with bosonic bath of infinite or finite degrees of freedom, while some have considered a qudit system as the environment. But in all of these studies, system-environment interaction has been considered to be homogeneous in space, and the issue of non-Markovian to Markovian transition in terms of system-environment coupling strength has not been addressed. Note that, non-Markovian to Markovian transition is, in general, not a trivial issue, as in most cases finite dimensional environments give rise to non-Markovianity.

In our study, we attempt to analyze the problem of whether a transition from non-Markovianity to Markovianity can be engineered for the spin bath model^33^. We particularly choose the spin bath model since it has wide ranging applications in simulating real physical scenarios^33–35^. In our attempt, we face a serious difficulty in diagonalizing the spin bath Hamiltonian, either analytically or numerically, for larger number of spins in the environment. Although, analytic solutions do exist for constant coupling^36^ and some special forms of time dependent coupling^37^, general solution for arbitrary forms of system-environment coupling of the spin bath Hamiltonian are hard to find. We therefore, try to circumvent the problem by choosing a simple model, which we argue, is a close approximation to the spin bath model for low temperatures. We choose an exchange type of interaction between a system qubit and individual environment qubits, where for each environment qubit the coupling can be chosen to be of different time dependent forms. But unlike the spin bath case, in our model, when the exchange interaction takes place between the system and a particular environment qubit, the rest of the environment qubits remain in a ground state; which also closely resembles the state of environment for low temperatures. As we will see in the paper, this approximation helps us to calculate and analyze non-Markovian to Markovian transition for different types of system-environment coupling.

We present four scenarios here, for different forms of system-environment coupling: (i) the coupling is time-independent and homogeneous over environment qubits, (ii) the coupling is time-independent but inhomogeneous over environment qubits, (iii) the coupling is homogeneous over the environment but is time-dependent, and (iv) the coupling is both time-dependent and inhomogeneous. We find that cases (i) and (ii) always give rise to non-Markovian system dynamics. For cases (iii) and (iv), we find that some functional forms of coupling for certain ranges of coupling strengths gives rise to non-Markovianity. For example in case (iii), polynomial forms of coupling always give rise to non-Markovian system dynamics, while exponential coupling give rise to non-Markovian system dynamics only for certain ranges of parameter values. In case (iv) we find that a cross-over from non-Markovianity to Markovianity can be achieved by varying the strength of coupling. We also calculate, the extremal values of coupling parameter beyond which non-Markovianity can no longer be detected. Thus we see, these extremal values act as critical values for transition from non-Markovian to Markovian regime. It is worth mentioning here that, for the purpose of detecting non-Markovianity we use Rivas-Huelga-Plenio (RHP) measure of non-Markovianity as proposed in^38^. Although there are different approaches of defining Markovianity and each approach represent different aspects of Markovianity, for the purpose of the present paper we choose, detection by the RHP measure as the definition of Markovianity.

Similar works on this line were done in^39–41^. But in the first approach^39^, the system qubit directly interacts with a single environment qubit and the rest of the environment qubits, only have an indirect effect on the system via the environment qubit directly attached. Also, the coupling parameters involved do not have any time dependence. In the second approach^40^, the transition from Markovianity to non-Markovianity was shown with a two tier environment; the first one being a multiple-spin system, while the second one was a bosonic bath. Also in^41^, the coupling between the system and individual environment qubits were constant in space and time. We take into account all these factors and present a detailed study of a spin environment and cover *all* the relevant cases.

In the background section, we discuss the relevant background required for following the techniques used in the paper. In the next section, we present our model, followed by a section, where we introduce different types of couplings and analyze them. Finally, we present the results of our analysis, before concluding in the last section.

## Background

In this section we present the relevant background of Markovian dynamics and the definitions used in the paper. We also describe the measure of entanglement for two-qubit systems, which will also be used to quantify non-Markovianity of our dynamics.

### Quantum Markovian dynamics

A discrete time stochastic process is called Markovian (Markov chain) if the state of the system at time *t*_*n*_ depends solely on the state of the system at time *t*_*n*−1_. This concept of Markov chain can be extended to the continuous time stochastic processes as well^2^. However, generalizing it to quantum dynamics is a difficult task. Numerous prescriptions have been proposed to capture different aspects of quantum Markovianity. Broadly these prescriptions can be classified into two classes: information backflow^42–47^ and completely positive divisibility (CP-divisibility)^1,48^.

#### Information backflow

The information backflow approach is inspired from the fact that a Markovian dynamics is characterized by unidirectional flow of information from the system to the environment. As for example, in the Lindblad master equation^49^, the non-negativity of the entropy production rate signifies unidirectional information flow from system to the environment, and thereby, is a signature of Markovianity. A dynamics is called Markovian from the information backflow approach, if some information quantifier decays over time in a monotonic way. Any departure from monotonicity of such quantifier is seen as a backflow of information from the environment, back to the system. Different quantifiers of information like distinguishability of states^42^, measure of entanglement^38^, quantum mutual information^44^, etc has also been suggested for this purpose. Each quantifier provides a different definition of Markovianity; all of which, are not in general equivalent. Only recently, there has been attempts to unify all these different definitions^45–47^ to provide a unified approach to information backflow.

#### CP-divisibility

Any dynamical process, given by completely-positive (CP) trace preserving (TP) map Λ_*t*_ representing evolution up to time *t* is called CP-divisible if

1 $${{\rm{\Lambda }}}_{t}={V}_{t,s}\,\circ \,{{\rm{\Lambda }}}_{s},$$

where *V*_*t*,*s*_ is CP for any *t* ≥ *s* ≥ 0, and ° denotes composition.

Although the most general description of Markovian dynamics is given by CP-Divisibility^1,48^, for our purpose we consider information backflow, in terms of measure of entanglement i e. the RHP measure, as the description of Markovianity.

### Detecting non-Markovianity through Entanglement

Let us first discuss entanglement measure of a two-qubit state. The entanglement between two two-level systems (two qubits) can be characterized by the Peres-Horodecki criterion^50,51^ which states that a two-qubit state *ρ*_as_, shared between a system qubit *s* and an ancilla qubit *a*, is entangled if and only if the partial transpose of this state, i.e. ${({\rho }_{{\rm{as}}})}^{{T}_{s}}$, is not a positive-semidefinite operator i.e. ${({\rho }_{as})}^{{T}_{s}}\,\ngeqq \,0$. Notably, for a two-qubit entangled state, the operator ${({\rho }_{{\rm{as}}})}^{{T}_{s}}$ has exactly one negative eigenvalue *λ*^52,53^. Thus |*λ*| may be used as a measure of entanglement for the state *ρ*_as_. Formally, the entanglement measure can be defined as follows

2 $$E({\rho }_{as})=|\lambda |=\frac{{\parallel {({\rho }_{as})}^{{T}_{s}}\parallel }_{1}-1}{2}$$

where, ${\Vert A\Vert }_{1}={\rm{Tr}}\sqrt{{A}^{\dagger }A}$ is the trace norm of a matrix *A*. Note that, *E*(*ρ*_*as*_) is nothing but the negativity of the bipartite state *ρ*_*as*_^54^. We will use this measure of entanglement as the quantifier for ascertaining Markovianity of the dynamics from the information backflow approach. Using entanglement to detect non-Markovianity was first used by Rivas, Huelga and Plenio in^38^, and this measure has been so called the RHP measure of non-Markovianity. Following their technique we attach an ancilla to the system, on which a dynamical map Λ_*t*_ is acting. Following the information backflow approach, the dynamical map Λ_*t*_ is called Markovian if $E(({\mathbb{1}}\otimes {{\rm{\Lambda }}}_{t})[|{{\rm{\Phi }}}^{+}\rangle \langle {{\rm{\Phi }}}^{+}|])$ is a monotonically non-increasing function of time *t*, where $|{{\rm{\Phi }}}^{+}\rangle \langle {{\rm{\Phi }}}^{+}|$ is the maximally entangled state, given by

3 $$|{{\rm{\Phi }}}^{+}\rangle =\frac{1}{\sqrt{2}}(|00\rangle +|11\rangle \mathrm{).}$$

## The Model

**Figure 1:**
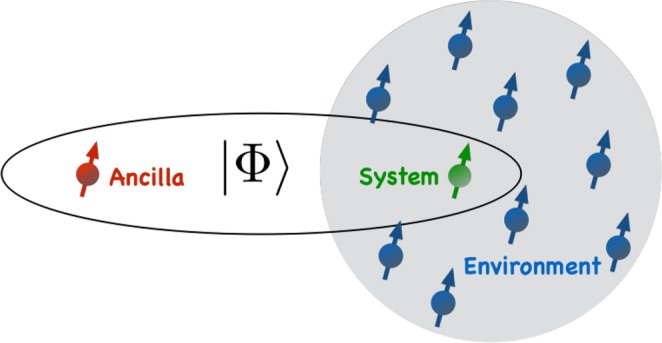
Schematic diagram of system qubit and ancilla qubit sharing a maximally entangled state $|{\rm{\Phi }}\rangle =\frac{1}{\sqrt{2}}(|11\rangle +|00\rangle )$. The system is interacting with an environment consisting of finite number of non-interacting qubits.

In this section, we present our model and discuss the motivation behind choosing it. We also describe the technique in detail, in which non-Markovianity in the system dynamics is detected. We consider two qubits, one of which is called the system (*s*) and the other, the ancilla (*a*). The system qubit is placed in an environment consisting of *N* non-interacting qubits (see Fig. 1). We take the interaction between the system qubit and the environment in the following form,

4 $$\begin{array}{ccc}{\mathop{H}\limits^{ \sim }}_{se}(t) & = & \hslash \alpha {[|1\rangle }_{s}\langle 0|\otimes \sum _{n=1}^{N}{\mathop{g}\limits^{ \sim }}_{n}^{\ast }(t)|{\mathrm{0..0}}_{n}..0{\rangle }_{e}\langle {\mathrm{0..1}}_{n}..0|\\  &  & +\,|0{\rangle }_{s}{\langle 1|\otimes \sum _{n=1}^{N}{\mathop{g}\limits^{ \sim }}_{n}(t)|{\mathrm{0..1}}_{n}..0\rangle }_{e}\langle {\mathrm{0..0}}_{n}..0|],\end{array}$$

where $|0\rangle$ and $|1\rangle$, respectively represent the ground and excited states of each qubit.

The coupling strength ${\tilde{g}}_{n}(t)$ is a complex number which can be time-dependent as well as site-dependent, and *α* is a real parameter with the dimension of frequency. The extra factor *α* is introduced to make the coupling strengths ${\tilde{g}}_{n}(t)$ dimensionless. For all practical purposes *α* can be assumed to be 1. The free Hamiltonians of the system and the environment are respectively given by,

5 $${H}_{s}=\frac{\hslash {\omega }_{s}}{2}{\sigma }_{z},$$

6 $${H}_{e}=\sum _{n}\frac{\hslash {\omega }_{n}}{2}{\sigma }_{z}^{(n)}\mathrm{.}$$

It is convenient to work in the interaction picture where we replace the total Hamiltonian $$H={H}_{s}+{H}_{e}+$$$${\tilde{H}}_{{\rm{se}}}\equiv {H}_{0}+{\tilde{H}}_{{\rm{se}}}$$ by the interaction picture Hamiltonian $${H}_{{\rm{se}}}(t)=\exp (i{H}_{0}t/\hslash ){\tilde{H}}_{{\rm{se}}}\,\exp (\,-\,i{H}_{0}t/\hslash )$$ which reads,

7 $$\begin{array}{ll}{H}_{{\rm{se}}}(t) & =\,\hslash \alpha [|1{\rangle }_{s}{\langle 0|\otimes \sum _{n=1}^{N}{\tilde{g}}_{n}^{\ast }(t){e}^{i\delta {\omega }_{n}t}|{\mathrm{0..0}}_{n}\mathrm{..0}\rangle }_{e}\langle {\mathrm{0..1}}_{n}\mathrm{..0}|\\  & \,+\,|0{\rangle }_{s}{\langle 1|\otimes \sum _{n\mathrm{=1}}^{N}{\tilde{g}}_{n}(t){e}^{-i\delta {\omega }_{n}t}|{\mathrm{0..1}}_{n}\mathrm{..0}\rangle }_{e}\langle {\mathrm{0..0}}_{n}\mathrm{..0}|]\\  & =\,\hslash \alpha {[|1\rangle }_{s}{\langle 0|\otimes \sum _{n=1}^{N}{g}_{n}^{\ast }(t)|{\mathrm{0..0}}_{n}\mathrm{..0}\rangle }_{e}\langle {\mathrm{0..1}}_{n}\mathrm{..0}|\\  & \,+\,|0{\rangle }_{s}{\langle 1|\otimes \sum _{n=1}^{N}{g}_{n}(t)|{\mathrm{0..1}}_{n}\mathrm{..0}\rangle }_{e}\langle {\mathrm{0..0}}_{n}\mathrm{..0}|]\end{array}$$

where *δω*_*n*_ = *ω*_*s*_ − *ω*_*n*_ and ${g}_{n}(t)={\tilde{g}}_{n}(t){e}^{-i\delta {\omega }_{n}}$. Henceforth, our discussion will be based on the Hamiltonian *H*_*se*_(*t*). We also consider the initial state of the environment to be in the thermal state,

8 $${\rho }_{e}\mathrm{(0)}=[p|0\rangle \langle 0|+\mathrm{(1}-p)|1\rangle {\langle 1|]}^{\otimes N},$$

where *p* = (1 + *e*^−*β*^)^−1^ and *β* is a positive real parameter which can be identified as the inverse of the temperature *T* of the environment.

### Motivation behind the model

Here we argue that, our model is a close approximation to the ‘spin bath’ model^34,35^ at low temperatures. Note that the Hamiltonian in Eq. (7) can also be written as,

9 $$\begin{array}{ccc}{H}_{{\rm{s}}{\rm{e}}}(t) & = & \hslash \alpha \sum _{n=1}^{N}\{{g}_{n}^{\ast }(t){\sigma }_{+}^{(s)}\otimes [|0\rangle \langle 0|\otimes ..{\sigma }_{-}^{(n)}..\otimes |0\rangle {\langle 0|]}_{e}\\  &  & +\,{g}_{n}(t){\sigma }_{-}^{(s)}\otimes [|0\rangle \langle 0|\otimes ..{\sigma }_{+}^{(n)}..\otimes |0\rangle {\langle 0|]}_{e}\}\end{array}$$

where ${\sigma }_{+}=|0\rangle \,\langle 1|$ and ${\sigma }_{-}=|1\rangle \langle 0|$. When we compare Eq. (9) with the usual Hamiltonian of a spin bath model^34–36^ in the interaction picture, given by,

10 $${H}_{spin-bath}=\hslash \alpha \sum _{n=1}^{N}({\sigma }_{x}^{(s)}{\sigma }_{x}^{(n)}+{\sigma }_{y}^{(s)}{\sigma }_{y}^{(n)}+{\sigma }_{z}^{(s)}{\sigma }_{z}^{(n)})$$

we find that the only difference comes from the |0〉 〈0| factors arising in Eq. (9), which are replaced by ${\mathbb{1}}$ for the spin bath Hamiltonian. As a result of this difference, the dynamics of the spin bath model is not entirely the same as our model. In the former, an exchange of one quanta of energy takes place between the system and individual environment qubit, when the rest of the environment qubits are allowed to be in any state, whereas in the later, the exchange will only take place when the rest of the environment qubits are in their ground state. This difference, although significant in general, will not play a major role when the state of the environment is close to the ground states, or in other words, temperature of the environment is low. Note that low temperature of environment correspond to values of *p* in Eq. (8), which are very close to 1, and this also confirms the fact that for low temperatures *ρ*_*e*_ is close to the ground state. Thus we see for low temperatures our model serves as a close approximation to the spin bath model. The main advantage of our model is the fact that our Hamiltonian is easily diagonalizable, and for certain types of couplings, as we discuss later in detail, allows for exact determination of the system dynamics in terms of Kraus operators, for *any* number of environment qubits.

We also stress that, although our model shows similarity to the spin bath model for low temperatures, we find solutions and analyze the dynamics of our model for any temperature whatsoever. The reason behind this is that our model being analytically solvable for certain types of couplings, allows for an opportunity to exactly solve the dynamics for any number of environment qubits, which is not often the case for systems with large number of spins. Note that, even for the spin-bath Hamiltonian, it is not easy to find the exact solution for non-zero temperatures.

### Diagonalizing the Hamiltonian of our model

There are only two non-zero eigenvalues of the Hamiltonian *H*_s*e*_(*t*) and they are,

11 $${\varepsilon }_{\pm }(t)=\pm \,\hslash \alpha \sqrt{\sum _{n=1}^{N}|{g}_{n}(t{)|}^{2}}=\pm \,\varepsilon (t),$$

corresponding to the eigenvectors,

12 $$|{\chi }_{\pm }(t){\rangle }_{se}=\frac{1}{\sqrt{2}}{[|1\rangle }_{s}\otimes |0{\rangle }_{e}^{\otimes N}\pm \,|\xi (t){\rangle }_{se}],$$

where $${|\xi (t)\rangle }_{se}={|0\rangle }_{s}\otimes {|{\beta }_{0}\rangle }_{e}$$ and,

13 $$|{\beta }_{0}{\rangle }_{e}=\frac{\hslash \alpha }{\varepsilon (t)}\sum _{n=1}^{N}{g}_{n}(t)|{\mathrm{0..1}}_{n}\mathrm{..0}{\rangle }_{e}\mathrm{.}$$

Thus, the time evolution operator *U*(*t*, 0) corresponding to the Hamiltonian *H*_s*e*_ is,

14 $$U(t,\,\mathrm{0)}={\mathscr{T}}\,\exp [-\frac{i}{\hslash }{\int }_{0}^{t}{H}_{se}(\tau ^{\prime} \mathrm{)\ }d\tau ^{\prime} ],$$

where ${\mathscr{T}}$ represents time ordering.

The ancilla qubit is used as a probe to characterize the non-Markovianity of the dynamics of the system in the presence of the environment. In order to do so we prepare the system and ancilla qubits in a maximally entangled state $|{{\rm{\Phi }}}^{+}\rangle$, as given in Eq. (3). Due to the interaction of the system qubit with the environment, the entanglement between the system and the ancilla qubit will evolve with time. The deviation of this time evolution of the entanglement, from monotonic decay is used to establish the non-Markovian character of the dynamics. Note here, that this idea was used by Rivas *et al*.^38^ to devise a measure of non-Markovianity. In the present paper, we follow this technique to consider the system dynamics to be non-Markovian whenever the entanglement between system and ancilla, as described above, shows non-monotonic behaviour, otherwise we consider the dynamics to be Markovian.

The joint initial state of the system plus ancilla plus environment is of the form,

15 $${\rho }_{{\rm{ase}}}\mathrm{(0)}=|{{\rm{\Phi }}}^{+}\rangle \langle {{\rm{\Phi }}}^{+}|\otimes {\rho }_{e}\mathrm{(0),}$$

which evolves to,

16 $${\rho }_{ase}(t)=[{{\mathbb{1}}}_{a}\otimes U(t,\,0)]{\rho }_{{\rm{a}}{\rm{s}}{\rm{e}}}(0)[{{\mathbb{1}}}_{a}\otimes {U}^{\dagger }(t,\,0)].$$

Therefore, reduced time-evolved system-ancilla state can be calculated by tracing out the environment part,

17 $${\rho }_{as}(t)={{\rm{Tr}}}_{e}\,{\rho }_{{\rm{ase}}}(t\mathrm{).}$$

## System - Environment Couplings

In this section, we introduce various classes of system-environment coupling, and in each case, we study their effect on the evolution of the system-ancilla joint state. We classify all the couplings into four major classes: (A) when the coupling parameter *g*_*n*_(*t*) is independent of the site index *n* (homogeneous) and time-independent; (B) when *g*_*n*_(*t*) is inhomogeneous but time-independent; (C) when *g*_*n*_(*t*) is homogeneous but time-dependent, and (D) when *g*_*n*_(*t*) is inhomogeneous and time-dependent. For each class, we calculate the entanglement of the time evolved state of system-ancilla, and thereby try to characterize the non-Markovian behaviour of the system dynamics. Henceforth, we assume *α* to be 1.

### Case A: Homogeneous and time-independent coupling

We have here the simplest situation, where the coupling of the system with all the environment qubits are uniform and time-independent i.e. *g*_*n*_(*t*) = *g*, a constant. As a result, the non-zero eigenvalues of the Hamiltonian, as given in Eq. (11), takes the form $${\varepsilon }_{\pm }(t)=\pm \,\varepsilon =$$$$\pm \,\hslash \sqrt{N}\mathrm{\ |}g|\equiv \hslash {\omega }_{0}$$, where $${\omega }_{0}=\sqrt{N}\mathrm{\ |}g|$$ is a constant with the dimension of frequency. The time-evolution operator *U*(*t*, 0) is of the form,

18 $$\begin{array}{rcl}U(t,0) & = & ({e}^{-i{\omega }_{0}t}-1)|{{\mathscr{X}}}_{+}\rangle \langle {{\mathscr{X}}}_{+}|\\  &  & +\,({e}^{i{\omega }_{0}t}-1)|{{\mathscr{X}}}_{-}\rangle \langle {{\mathscr{X}}}_{-}|+{\mathbb{1}}.\end{array}$$

Using the above form and the form of *ρ*_*e*_ given in Eq. (8), we find the Kraus operators *K*_*mn*_(*t*) of system dynamics, which are defined in the following way,

19 $${\rho }_{s}\mathrm{(0)}\to {\rho }_{s}(t)=\sum _{m,n=1}^{N}{K}_{mn}(t\mathrm{)\ }{\rho }_{s}\mathrm{(0)\ }{K}_{mn}^{\dagger }(t),$$

where the *N*^2^ Kraus operators are given by,

20 $${K}_{mn}(t)=\sqrt{{p}^{N-{s}_{n}}{\mathrm{(1}-p)}^{{s}_{n}}}[\begin{array}{cc}\frac{\cos \,{\omega }_{0}t-1}{{\omega }_{0}^{2}}{g}_{N-\mathrm{log}m}(t\mathrm{)\ }{g}_{N-\,\mathrm{log}\,n}^{\ast }(t)+{\delta }_{mn} & -\frac{i}{{\omega }_{0}}\,\sin \,{\omega }_{0}t\,{g}_{N-logm}(t)\,{\delta }_{0n}\\ -\frac{i}{{\omega }_{0}}\,\sin \,{\omega }_{0}t\,{g}_{N-\,\mathrm{log}\,n}^{\ast }(t)\,{\delta }_{0m} & (\cos \,{\omega }_{0}t-\mathrm{1)}\,{\delta }_{0m}{\delta }_{0n}+{\delta }_{mn}\end{array}]$$

where *m*, *n* = 1, …, *N*, log *x* refers to ${\mathrm{log}}_{2}\,x$, and *s*_*n*_ is the number of 1’s in the binary equivalent of *n*. For example, if *n* = 6, then the binary equivalent of *n* is 110. Therefore *s*_*n*_ = 2.

We then find time evolved state of the system-ancilla, using Eqs (15–17),

21 $$\begin{array}{ccc}{\rho }_{{\rm{a}}{\rm{s}}}(t) & = & |{{\rm{\Phi }}}^{+}\rangle \langle {{\rm{\Phi }}}^{+}|-\frac{1}{2}[p{\kappa }_{0}(|11\rangle \langle 11|-|10\rangle \langle 10|)\\  &  & +\,(1-p){\kappa }_{0}(|00\rangle \langle 00|-|01\rangle \langle 01|)\\  &  & +\,{\delta }_{0}(|00\rangle \langle 11|+|11\rangle \langle 00|)],\end{array}$$

where ${\kappa }_{0}={p}^{N-1}{\sin }^{2}({\omega }_{0}t)$ and ${\delta }_{0}=2{p}^{N-1}{\sin }^{2}(\frac{{\omega }_{0}t}{2})$. The only possible negative eigenvalue of ${[{\rho }_{{\rm{as}}}(t)]}^{{T}_{s}}$, if any, is of the form,

22 $$\lambda (t)=\frac{1}{4}[{\kappa }_{0}-\sqrt{{\mathrm{(1}-2p)}^{2}{\kappa }_{0}^{2}+\mathrm{4(1}-{\delta }_{0}{)}^{2}}]\mathrm{.}$$

We present the plot of *E*(*ρ*_*a*_*s*(*t*)) =| *λ*(*t*)| versus time, later in the Result section.

### Case B: Inhomogeneous and time-independent coupling

Consider a system, where a single two-level system (perhaps an ion as an impurity) is placed in a spin lattice. The lattice sites, closest to the impurity interacts very strongly with the system, while, as we go away from the impurity site, the strength of interaction becomes weaker and weaker. In such cases, we have a scenario similar to our model, and the interaction parameter *g*_*n*_(*t*) is inhomogeneous, but there is no explicit time dependence. Therefore, *g*_*n*_(*t*) = *g*_*n*_. Hence, *ε*_±_(*t*) = ±*ε* = ℏ*ω* in Eq. (11) are also time-independent. Note, in this case also $\omega =\sqrt{{\sum }_{n\mathrm{=1}}^{N}|{g}_{n}{|}^{2}}$ is a constant with dimensions of frequency. Following this, the analysis is same as in the last subsection. As a result, the evolution operator *U*(*t*, 0), the time evolved state *ρ*_as_(*t*) and the only possible negative eigenvalue, if any, of ${[{\rho }_{{\rm{as}}}(t)]}^{{T}_{s}}$ for this case are of the same forms as in Eqs (18, 20–22) respectively, except for *ω*_0_, in appropriate places, replaced by *ω*.

### Case C: Homogeneous and time-dependent coupling

So far we have considered only couplings which are independent of time. In this section, we consider time-dependent and homogeneous couplings. We take an *arbitrary* real function of time, which is independent of site index *n* i.e. *g*_*n*_(*t*) = *g*(*t*). Note that our coupling operator between system and individual environment qubit, as given in Eq. (7), is of the form *σ*_+_ ⊗ *σ*_−_ + *σ*_−_ ⊗ *σ*_+_ which can also be expressed as *σ*_*x*_ ⊗ *σ*_*x*_ + *σ*_*y*_ ⊗ *σ*_*y*_. Thus, our system-environment coupling is a special case of the *XY* coupled Hamiltonian. Such coupling with time-dependent coefficients have been used to show non-trivial entanglement dynamics^55,56^.

Fortunately, the Hamiltonians *H*_*se*_(*t*) in this case commutes at different times, which makes the analysis similar to the one in case B. The only difference being the non-zero eigenvalues, in Eq. (11), to be of the form ${\varepsilon }_{\pm }(t)=\pm \,\hslash \sqrt{N}|g(t)|=\pm \,\varepsilon (t)$, which is no longer constant in time. The whole treatment of the dynamics of the system and the ancilla remains the same if we replace *ω*_0_ and *ω*_0_*t*, in Eqs (18, 20–22), by $\sqrt{N}\mathrm{\ |}g(t)|$ and Ω(*t*), respectively, where,

23 $$\begin{array}{rcl}{\rm{\Omega }}(t) & = & \frac{1}{\hslash }{\int }_{0}^{t}\varepsilon (\tau )d\tau ={\int }_{0}^{t}\,{(\sum _{n\mathrm{=1}}^{N}|{g}_{n}(\tau {)|}^{2})}^{\frac{1}{2}}d\tau \\  & = & \sqrt{N}{\int }_{0}^{t}|g(\tau )|d\tau \mathrm{.}\end{array}$$

### Case D: Inhomogeneous and time-dependent coupling

The most general class of coupling *g*_*n*_(*t*) is when it depends on both the site *n* and time *t*. The interaction Hamiltonian in such a situation does not commute at different times and this makes the calculation for solving the dynamics difficult. However, we can use numerical methods to simulate the time-evolution and get the solution for *ρ*_as_(*t*). One can obtain the following results analytically before starting the simulation part.

#### Analytical Part

Two of the eigenvalues of the Hamiltonian in Eq. (7) are non-zero, as given in Eq. (11). The remaining (2^*N*+1^ − 2) of the eigenvalues are zero. A possible choice for these null space eigenvectors are found in the following way:

**Step I:** We first feed the eigenvectors (corresponding to non-zero eigenvalues) given in Eq. (12) as rows of a matrix *A*. Note, *A* is a 2 × 2^(*N*+1)^ matrix

**Step II:** By row reduction method^57^, we find out a basis ${\mathcal B}$ for the Null space of *A*. Note that ${\mathcal B}$ is not necessarily ortho-normal.

#### Simulation Part

Obtaining an orthonormal basis ${\mathcal B} ^{\prime}$ from ${\mathcal B}$ analytically is a challenging job. We, therefore resort to numerical techniques for this case.

**Step I:** From ${\mathcal B}$, using Gram-Schmidt Orthonormalization procedure^57^, we find an orthonormal basis ${\mathcal B} ^{\prime}$. Note, ${\mathcal B} ^{\prime}$ forms the set of eigenvectors of the Hamiltonian, corresponding to zero eigenvalues.

**Step II:** As, the eigenvectors are time-dependent, the Hamiltonian is not different-time commuting. Hence, the evolution operator may be found numerically from the following expression,

24 $$\begin{array}{l}{U}_{se}(t,\,0)={\mathscr{T}}\,exp[-\frac{i}{\hslash }{\int }_{0}^{t}{H}_{se}(\tau ^{\prime} \mathrm{)\ }d\tau ^{\prime} ]\\ \,=\mathop{\mathrm{lim}}\limits_{m\to \infty }[exp[-\frac{i}{\hslash }{\int }_{(m-\mathrm{1)}\tau }^{m\tau }{H}_{se}(\tau ^{\prime} \mathrm{)\ }d\tau ^{\prime} ]\\ \,\times exp\,[-\frac{i}{\hslash }{\int }_{(m-\mathrm{2)}\tau }^{(m-\mathrm{1)}\tau }{H}_{se}(\tau ^{\prime} \mathrm{)\ }d\tau ^{\prime} ]\times \ldots \\ \,\times exp\,[-\frac{i}{\hslash }{\int }_{0}^{\tau }{H}_{se}(\tau ^{\prime} \mathrm{)\ }d\tau ^{\prime} ]],\end{array}$$

where ${\mathscr{T}}$ represents time ordering.

**Step III:** We evolve the initial ancilla -system-environment state *ρ*_*ase*_(0) by the unitary operator ${U}_{ase}(t,\,\mathrm{0)}={{\mathbb{I}}}_{a}\otimes {U}_{se}(t,\,\mathrm{0)}$ and get the time evolved state *ρ*_*ase*_(*t*).

**Step IV:** We trace out the environment from *ρ*_*ase*_(*t*) and get *ρ*_*as*_(*t*) = *Tr*_*e*_[*ρ*_*ase*_(*t*)]. We then evaluate our entanglement measure *E*(*t*) given in Eq. (2), on *ρ*_*as*_(*t*) and plot it as a function of time.

## Results

**Figure 2:**
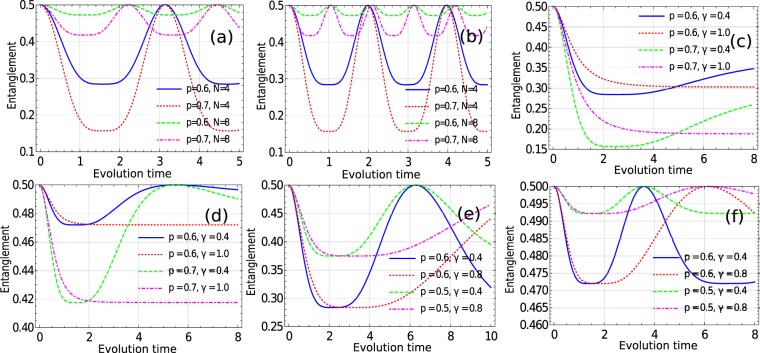
Plots showing the system-ancilla entanglement dynamics in different scenarios. For simplicity we have considered *α* = 1. (**a**) When the coupling is homogeneous and time-independent i.e. *g* = 1. (**b**) When the coupling is inhomogeneous and time-independent i.e. ${g}_{n}=\sqrt{n}$. (**c**) When the coupling is homogeneous and time-dependent, i.e. *g*_*n*_(*t*) = *g*(*t*) = exp(−*γt*) and *N* = 4. (**d**) Coupling is *g*(*t*) = exp(−*γt*) and *N* = 8. (**e**) Coupling is of the form, $g(t)=\frac{1}{1+\gamma t}$ and *N* = 4. (**f**) Coupling is $g(t)=\frac{1}{1+\gamma t}$ and *N* = 8.

**Figure 3:**
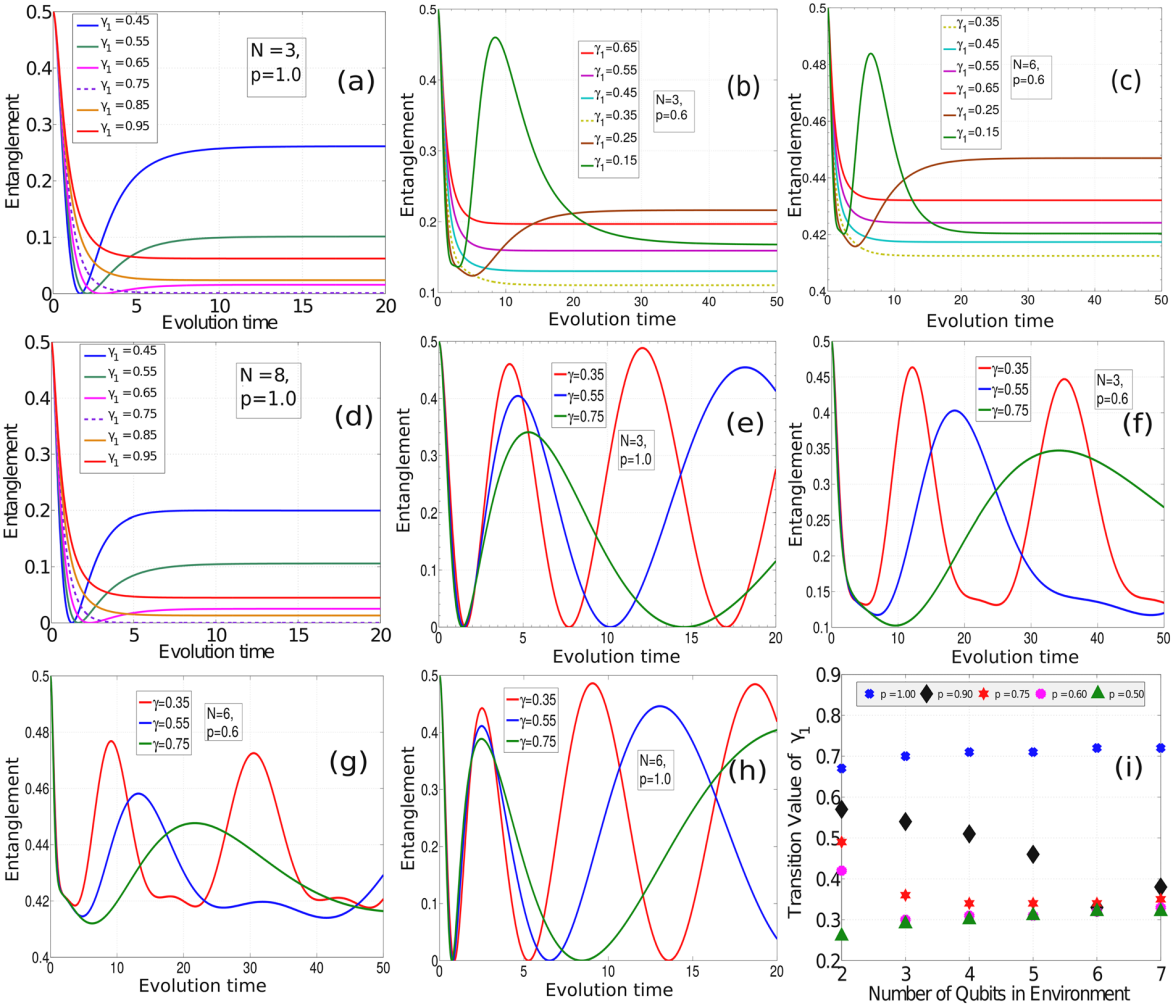
Plots showing the system-ancilla entanglement dynamics in different scenarios. For simplicity we have considered *α* = 1. (**a**–**d**) When the coupling is inhomogeneous and time-dependent i.e. ${g}_{n}(t)={e}^{-{\gamma }_{1}nt}$. (**e**–**h**) Coupling is ${g}_{n}(t)=\frac{1}{1+{t}^{n\gamma }}$. (**i**) Transition values of *γ*_1_ for coupling ${g}_{n}(t)={e}^{-{\gamma }_{1}nt}$ as a function of *N* for different values of *p*.

In this section, we show that some of the classes of the couplings that we have considered, always results in non-Markovian dynamics. However, there are also some classes for which we can tune the parameters and find a transition from non-Markovian to Markovian dynamics. In order to do so, we plot the entanglement dynamics between the system and ancilla, for each class, as a function of time and observed if there is any departure from monotonicity with time. As mentioned in Sec. III, this technique helps in characterizing non-Markovianity present in the system dynamics. In Figs 2 and 3, we present entanglement dynamics for different classes of the system-environment coupling, considered in the previous section. Also in Table 1, we provide a concise summary of all the resuts obtained in this section. We now present our findings for each class of system-environment coupling.

**Table 1:** Nature of dynamics for different forms of coupling.

Coupling	Value of p	Value of N	Parameter values	Nature
Homogeneous and time-independent	any value	any value	—	non-Markovian
Inhomogeneous and time-independent	any value	any value	—	non-Markovian
$g(t)={\sum }_{m}{c}_{m}{t}^{m}$	any value	any value	—	non-Markovian
*g*(*t*) = *e*^−*γt*^	any value	any value	*Re*(*γ*) ≤ 0, $Re(\gamma ) > \alpha \sqrt{N}/\pi$	non-Markovian
$g(t)=\frac{1}{1+\gamma \,t}$	0.6	4	*γ* = 0.4, 0.8	non-Markovian
	0.5	4	*γ* = 0.4, 0.8	
	0.6	8	*γ* = 0.4, 0.8	
	0.5	8	*γ* = 0.4, 0.8	
${g}_{n}(t)={e}^{-{\gamma }_{1}nt}$	1.0	3	*γ*1 = 0.45, ‥, 0.95	transition
	0.6	3	*γ*1 = 0.15, ‥, 0.65	from
	0.6	6	*γ*1 = 0.15, ‥, 0.35	non-Markovianity
	1.0	8	*γ*1 = 0.45, ‥, 0.95	to Markovianity
${g}_{n}(t)=\frac{1}{1+{t}^{n\gamma }}$	1.0	3	*γ* = 0.35, 0.55, 0.75	non-Markovian
	0.6	3	*γ* = 0.35, 0.55, 0.75	
	0.6	6	*γ* = 0.35, 0.55, 0.75	
	1.0	6	*γ* = 0.35, 0.55, 0.75	

### Cases A and B

In Fig. 2(a,b), we plot the entanglement as a function of time for the homogeneous time-independent, and the inhomogeneous time-independent couplings, respectively, i.e, *g*_*n*_(*t*) = *g* and *g*_*n*_(*t*) = *g*_*n*_, respectively. Note here, that *g* and *g*_*n*_, for all values of *n*, are arbitrary complex functions. Analytic calculations for the entanglement measure, as given in Eq. (22) suggests a periodic behaviour for both the classes, which can also be seen in Fig. 2(a,b). As a result, we conclude in both of these classes of couplings, the dynamics is always non-Markovian.

### Case C

We consider homogeneous and time-dependent couplings and find that if *g*(*t*) is some polynomial function of *t*, we will get Ω(*t*) as a polynomial function of t. This gives rise to a periodic function *λ*(*t*). As a result, the dynamics is non-Markovian, in general. Interestingly, it was recently pointed out^58^, that the Hamiltonian dialation obtained from the Choi state of the system dynamics, diverges whenever the system dynamics is time-independent Markovian. Although, in our case, the system-environment Hamiltonian (equivalent to the dialated Hamiltonian)is not related to the Choi state of the system dynamics in the same way as in^58^. As a result the conclusion of^58^ differs from ours. If *g*(*t*) = exp(−*γt*) then non-Markovianity can be witnessed if the real part *γ*_*r*_ of *γ* fails to be positive or violates the inequality $\alpha \sqrt{N}\ge {\gamma }_{r}\pi$. Figure 2(c,d) show the entanglement vs time plot for two values of *γ*_*r*_; one of which violates the above mentioned inequality. We also consider the case $g(t)=\frac{1}{1+\gamma t}$, and show in Fig. 2(e,f) that the dynamics is non-Markovian for various values of *γ*. *N* and *p*.

### Case D

For inhomogeneous time-dependent couplings, the dynamics can be made both non-Markovian and Markovian by choosing the strength of the coupling appropriately. We consider two special cases of inhomogeneous time-dependent coupling: (*i*) ${g}_{n}(t)={e}^{-{\gamma }_{1}nt}$, and (*ii*) ${g}_{n}(t)=\frac{1}{1+{t}^{n\gamma }}$. For simplicity, we have assumed *α* = 1. For analyzing coupling (*i*), we plot the system-ancilla entanglement measure as a function of time in Fig. 3(a–d), for different values of the coupling parameter *γ*_1_, at a fixed values of *N* and *p*. In Fig. 3(a–d), monotonically decreasing entanglement values show signs of Markovianity and non-monotonic decay are evidence of non-Markovianity. As expected, increasing the coupling parameter *γ*_1_ i.e., decreasing coupling strength, leads to the transition from non-Markovian to Markovian dynamics. The figures also show an interesting feature that, after sufficient time, the entanglement in the system-ancilla state saturates to fixed values irrespective of their Markovian or non-Markovian nature. This feature can be signs of possible equilibration of the system ancilla state. Next, we find the extremal values of *γ*_1_ for which non-Markovianity is witnessed. These extremal values serve as transition parameters from non-Markovianity to Markovianity. On plotting these transition values as a function of *N* (see Fig. 3(i)), it appears that a saturation is reached as *N* is increased for values *p* = 0.5 and *p* = 1.0. We perceive, this is the result of the fact that for this type of coupling i.e. ${g}_{n}(t)={e}^{-{\gamma }_{1}nt}$, the larger is the value of *N*, the smaller is its effect on the system dynamics. Although a definitive conclusion about whether the saturation persists over large *N* can only be made after computing the transition values for larger values of *N*, it is a computationally demanding process with the computational facilities available at our disposal. For coupling (*ii*), the dynamics shows non-Markovianity for various values of *γ*, *N* and *p*, as shown in Fig. 3(e–h).

## Conclusion

In this paper, we have addressed the question of how non-Markovianity of a dynamics changes with the interaction between the system and the environment and also with size of the environment. We have taken a simple model constituting of a few qubits, which can also be seen as a close approximation to the spin bath model, for low temperatures. Even in this minimalistic scenario, we were able to find a transition from non-Markovian to Markovian dynamics by tuning the system-environment interaction. This is somewhat counterintuitive as it is generally conceived that for having Markovian dynamics the bath/environment should have infinite degrees of freedom, although there are exceptions^59^. We also found, in our model, that if the interaction Hamiltonian is time-independent, the dynamics is always non-Markovian, irrespective of the size of environment. Note that in this scenario for a general interaction Hamiltonian in the weak coupling limit, we generally get to see Markovian dynamics only for a very large size of bath; at least in the case of harmonic oscillator bath. The present scenario is different as we have considered spin environment and the system-environment interaction is of very specific type. In the case of site-independent interaction, polynomial forms and certain cases of exponential forms of interaction show non-Markovianity. Lastly, we study time-dependent and site-dependent interaction for certain forms of system-environment coupling. In this last case, we also saw a transition from non-Markovian to Markovian regime. Interestingly, the transition values appear to saturate to a certain value depending on the initial temperature of the environment, as the number of environment qubits increases. Examining this type of spin environment is recently drawing some amount of interest^33^. Studies on similar lines was also done recently in^36^, where an analysis of a qubit system interacting with a sea of spins was given. A number of questions arise from the present study: whether such a transition can be found by considering more general forms of interaction, what happens if along with system-environment interaction there is some interaction present among the environment particles themselves, etc. One may also find it useful to check, whether the aforesaid saturation of transition parameters is a general feature of interaction that exhibits non-Markovian to Markovian transition.

## References

[1] Breuer H-P, Laine E-M, Piilo J, Vacchini B (2016). Colloquium: Non-markovian dynamics in open quantum systems. Reviews of Modern Physics.

[2] Rivas A, Huelga SF, Plenio MB (2014). Quantum non-markovianity: characterization, quantification and detection. Reports on Progress in Physics.

[3] Bylicka, B., Chruściński, D. & Maniscalco, S. Non-markovianity and reservoir memory of quantum channels: a quantum information theory perspective. *Scientific reports* **4** (2014). 10.1038/srep05720 PMC4104480 25043763

[4] Man, Z.-X., Xia, Y.-J. & Franco, R. L. Cavity-based architecture to preserve quantum coherence and entanglement. *Scientific reports* **5** (2015). 10.1038/srep13843 PMC4563358 26351004

[5] Franco RL (2016). Nonlocality threshold for entanglement under general dephasing evolutions: a case study. Quantum Information Processing.

[6] Aolita L, De Melo F, Davidovich L (2015). Open-system dynamics of entanglement: a key issues review. Reports on Progress in Physics.

[7] Mortezapour A, Franco RL (2018). Protecting quantum resources via frequency modulation of qubits in leaky cavities. Scientific reports.

[8] Orieux A (2015). Experimental on-demand recovery of entanglement by local operations within non-markovian dynamics. Scientific reports.

[9] Xu J-S (2010). Experimental demonstration of photonic entanglement collapse and revival. Phys. Rev. Lett..

[10] de Vega I, Alonso D (2017). Dynamics of non-markovian open quantum systems. Reviews of Modern Physics.

[11] Man Z-X, Xia Y-J, Franco RL (2018). Temperature effects on quantum non-markovianity via collision models. Physical Review A.

[12] Mortezapour A, Naeimi G, Franco RL (2018). Coherence and entanglement dynamics of vibrating qubits. Optics Communications.

[13] Mortezapour A, Borji MA, Franco RL (2017). Protecting entanglement by adjusting the velocities of moving qubits inside non-markovian environments. Laser Physics Letters.

[14] Mortezapour A, Borji MA, Park D, Franco RL (2017). Non-markovianity and coherence of a moving qubit inside a leaky cavity. Open Systems & Information Dynamics.

[15] Franco RL, D’Arrigo A, Falci G, Compagno G, Paladino E (2014). Preserving entanglement and nonlocality in solid-state qubits by dynamical decoupling. Physical Review B.

[16] Bellomo B, Compagno G, Lo Franco R, Ridolfo A, Savasta S (2011). Dynamics and extraction of quantum discord in a multipartite open system. International Journal of Quantum Information.

[17] Dijkstra AG, Tanimura Y (2010). Non-markovian entanglement dynamics in the presence of system-bath coherence. Physical review letters.

[18] D’Arrigo A, Franco RL, Benenti G, Paladino E, Falci G (2014). Recovering entanglement by local operations. Annals of Physics.

[19] Fanchini, F. F., Pinto, D. d. O. S. & Adesso, G. *Lectures on General Quantum Correlations and Their Applications* (Springer, 2017).

[20] Bellomo B, Franco RL, Andersson E, Cresser JD, Compagno G (2012). Dynamics of correlations due to a phase-noisy laser. Physica Scripta.

[21] González-Gutiérrez CA, Román-Ancheyta R, Espitia D, Lo Franco R (2016). Relations between entanglement and purity in non-markovian dynamics. International Journal of Quantum Information.

[22] Xu, J.-S. *et al*. Experimental recovery of quantum correlations in absence of system-environment back-action. *Nature communications* **4** (2013). 10.1038/ncomms3851 PMC3868330 24287554

[23] Franco RL, Bellomo B, Maniscalco S, Compagno G (2013). Dynamics of quantum correlations in two-qubit systems within non-markovian environments. International Journal of Modern Physics B.

[24] Schmidt S (1999). Non-markovian effects in strong-field pair creation. Physical Review D.

[25] Bloch JC, Roberts CD, Schmidt S (2000). Memory effects and thermodynamics in strong field plasmas. Physical Review D.

[26] Rebentrost, P. & Aspuru-Guzik, A. Communication: Exciton–phonon information flow in the energy transfer process of photosynthetic complexes (2011). 10.1063/1.3563617 21405149

[27] Liu B-H (2011). Experimental control of the transition from markovian to non-markovian dynamics of open quantum systems. Nature Physics.

[28] Bernardes N, Carvalho A, Monken C, Santos MF (2014). Environmental correlations and markovian to non-markovian transitions in collisional models. Physical Review A.

[29] Brito F, Werlang T (2015). A knob for markovianity. New Journal of Physics.

[30] Garrido N, Gorin T, Pineda C (2016). Transition from non-markovian to markovian dynamics for generic environments. Physical Review A.

[31] Man Z-X, Xia Y-J, Franco RL (2015). Harnessing non-markovian quantum memory by environmental coupling. Physical Review A.

[32] Franco RL (2015). Switching quantum memory on and off. New Journal of Physics.

[33] Prokof’ev N, Stamp P (2000). Theory of the spin bath. Reports on Progress in Physics.

[34] Hutton A, Bose S (2004). Mediated entanglement and correlations in a star network of interacting spins. Physical Review A.

[35] Breuer H-P, Burgarth D, Petruccione F (2004). Non-markovian dynamics in a spin star system: Exact solution and approximation techniques. Physical Review B.

[36] Bhattacharya S, Misra A, Mukhopadhyay C, Pati AK (2017). Exact master equation for a spin interacting with a spin bath: Non-markovianity and negative entropy production rate. Physical Review A.

[37] Jing J, Wu L-A (2018). Decoherence and control of a qubit in spin baths: an exact master equation study. Scientific reports.

[38] Rivas Á, Huelga SF, Plenio MB (2010). Entanglement and non-markovianity of quantum evolutions. Physical review letters.

[39] Apollaro TJ, Di Franco C, Plastina F, Paternostro M (2011). Memory-keeping effects and forgetfulness in the dynamics of a qubit coupled to a spin chain. Physical Review A.

[40] Lorenzo S, Plastina F, Paternostro M (2013). Tuning non-markovianity by spin-dynamics control. Physical Review A.

[41] Wang Z, Guo Y, Zhou D (2013). Non-markovian dynamics in a spin star system: the failure of thermalisation. The European Physical Journal D.

[42] Breuer H-P, Laine E-M, Piilo J (2009). Measure for the degree of non-markovian behavior of quantum processes in open systems. Physical review letters.

[43] Wißmann S, Breuer H-P, Vacchini B (2015). Generalized trace-distance measure connecting quantum and classical non-markovianity. Physical Review A.

[44] Luo S, Fu S, Song H (2012). Quantifying non-markovianity via correlations. Physical Review A.

[45] Bylicka B, Johansson M, Acn A (2017). Constructive method for detecting the information backflow of non-markovian dynamics. Physical review letters.

[46] Chruściński D, Rivas Á, Størmer E (2018). Divisibility and information flow notions of quantum markovianity for noninvertible dynamical maps. Physical review letters.

[47] Chakraborty S (2018). Generalized formalism for information backflow in assessing markovianity and its equivalence to divisibility. Physical Review A.

[48] Chruściński D, Kossakowski A (2012). Markovianity criteria for quantum evolution. Journal of Physics B: Atomic, Molecular and Optical Physics.

[49] Breuer, H.-P. & Petruccione, F. *The theory of open quantum systems* (Oxford University Press on Demand, 2002).

[50] Peres A (1996). Separability criterion for density matrices. Physical Review Letters.

[51] Horodecki M, Horodecki P, Horodecki R (1996). Separability of mixed states: necessary and sufficient conditions. Physics Letters A.

[52] Sanpera A, Tarrach R, Vidal G (1998). Local description of quantum inseparability. Physical Review A.

[53] Rana S, Parashar P (2012). Entanglement is not a lower bound for geometric discord. Physical Review A.

[54] Vidal G, Werner RF (2002). Computable measure of entanglement. Physical Review A.

[55] Sadiek G, Alkurtass B, Aldossary O (2010). Entanglement in a time-dependent coupled xy spin chain in an external magnetic field. Physical Review A.

[56] Bortz M, Stolze J (2007). Spin and entanglement dynamics in the central-spin model with homogeneous couplings. Journal of Statistical Mechanics: Theory and Experiment.

[57] Hoffman, K. & Kunze, R. Linear algebra, 2nd (1990).

[58] Dive B, Mintert F, Burgarth D (2015). Quantum simulations of dissipative dynamics: Time dependence instead of size. Physical Review A.

[59] Pang, S., Brun, T. A. & Jordan, A. N. Abrupt transitions between markovian and non-markovian dynamics in open quantum systems. *arXiv preprint arXiv:1712.10109* (2017).

