# Author Correction: Non-Markovianity of qubit evolution under the action of spin environment

**DOI:** 10.1038/s41598-019-55327-z

**Published:** 2019-12-06

**Authors:** Sagnik Chakraborty, Arindam Mallick, Dipanjan Mandal, Sandeep K. Goyal, Sibasish Ghosh

**Affiliations:** 1 Optics and Quantum Information Group, The Institute of Mathematical Sciences, C. I. T. Campus, Taramani, Chennai, 600113 India; 2 Homi Bhabha National Institute, Training School Complex, Anushakti Nagar, Mumbai, 400094 India; 3 The Institute of Mathematical Sciences, C. I. T. Campus, Taramani, Chennai, 600113 India; 4 Department of Physics, Indian Institute of Science Education and Research, Mohali, Punjab 140306 India

Correction to: *Scientific Reports* 10.1038/s41598-019-39140-2, published online 27 February 2019

This Article contains errors in the equation and text under subheading ‘Motivation behind the model’.

“When we compare Eq. (9) with the usual Hamiltonian of a spin bath model^34,35,36^ in the interaction picture, given by,

$${H}_{spin-bath}=\hslash \alpha \mathop{\sum }\limits_{n=1}^{N}({\sigma }_{x}^{(s)}{\sigma }_{x}^{(n)}+{\sigma }_{y}^{(s)}{\sigma }_{y}^{(n)}+{\sigma }_{z}^{(s)}{\sigma }_{z}^{(n)})$$

we find that the only difference comes from the |0〉 〈0| factors arising in Eq. (9), which are replaced by 𝟙 for the spin bath Hamiltonian.”

should read:

“When we compare Eq. (9) with the usual Hamiltonian of a spin bath model^34,35^ in the interaction picture, given by,

$${H}_{spin-bath}=\hslash \alpha \mathop{\sum }\limits_{n=1}^{N}({\sigma }_{x}^{(s)}{\sigma }_{x}^{(n)}+{\sigma }_{y}^{(s)}{\sigma }_{y}^{(n)})$$

we find that the only difference comes from the |0〉 〈0| factors arising in Eq. (9), which are replaced by 𝟙 for the spin bath Hamiltonian.”

Additionally, the Acknowledgements section in this Article is incomplete.

“The authors would like to thank R. Lo Franco, C. Gonzlez-Gutirrez, F. Plastina and T.J.G. Apollaro for providing useful inputs on the manuscript. SC, AM, and SG gratefully acknowledge discussions with Samyadev Bhattacharya and Chiranjib Mukhopadhyay.”

should read:

“The authors would like to thank R. Lo Franco, C. Gonzlez-Gutirrez, F. Plastina and T.J.G. Apollaro for providing useful inputs on the manuscript. SC, AM, and SG gratefully acknowledge discussions with Samyadev Bhattacharya and Chiranjib Mukhopadhyay.

SC, AM, DM and SG acknowledges The Institute of Mathematical Sciences for financial support. SKG acknowledges the financial support from SERB-DST (File no. ECR/2017/002404).”

